# Blood–Brain Barrier and Neuronal Model Systems for Studying CoQ_10_ Metabolism

**DOI:** 10.3390/antiox15010041

**Published:** 2025-12-28

**Authors:** David Mantle, Neve Cufflin, Mollie Dewsbury, Iain Parry Hargreaves

**Affiliations:** 1 Pharma Nord (UK) Ltd., Morpeth NE61 2DB, Northumberland, UK; 2 School of Pharmacy and Biomolecular Sciences, Liverpool John Moores University, Liverpool L3 5UX, Merseyside, UK or i.hargreaves@ucl.ac.uk (I.P.H.)

**Keywords:** coenzyme Q10, vitamin E, selenium, blood–brain barrier, 2D and 3D model systems, intranasal, STARD7

## Abstract

The disparity in outcomes between preclinical and clinical studies supplementing coenzyme Q10 (CoQ10) in neurological disorders may be a reflection of the differences in the ability of supplemental CoQ10 to access the blood–brain barrier (BBB) in rodents and in humans, which is, in turn, a consequence of contrasting structures of the BBB. The applicability of in vivo animal models to study access of CoQ10 across the BBB and subsequent neuronal metabolism has, therefore, been questioned, and there is an argument, perhaps surprisingly, that in vitro model systems (particularly 3D cellular systems) may be more appropriate. In this article, we have, therefore, reviewed the role of model systems to study the access of CoQ10 across the BBB, as well as the role of such systems in studying the role of CoQ10 in aspects of neuronal metabolism, such as mitochondrial and lysosomal function. In addition, the use of such model systems to study the interactions of CoQ10 with vitamin E and selenium has been reviewed. Finally, the practical application of a neuronal model system to investigate the effect of CoQ10 supplementation on CoQ10 status and mitochondrial metabolism in a CoQ10 deficiency state has been described.

## 1. Introduction

Clinical studies supplementing coenzyme Q10 (CoQ10) in disorders such as heart failure have reported significant benefit [1]; however, the outcomes of clinical trials supplementing CoQ10 in neurological disorders have, in general, been disappointing. Thus, while promising results have been obtained from preclinical studies supplementing CoQ10 in animal models of Parkinson’s disease, Alzheimer’s disease, or amyotrophic lateral sclerosis, corresponding randomised controlled trials in these disorders have reported no significant benefit [2]. This disparity in outcomes between preclinical and clinical studies may be a reflection of the disparity in the ability of supplemental CoQ10 to access the blood–brain barrier (BBB) in rodents and humans, which is, in turn, a consequence of corresponding structural differences. It is of note that in primary CoQ10 deficiency, patients affected in non-CNS tissues (particularly skeletal muscle and kidney) may respond well to supplementation with CoQ10, whereas, in contrast, patients with neurological symptoms in general do not respond well to treatment [3]; this, in turn, suggests that CoQ10 has difficulty crossing the BBB in humans. The applicability of in vivo animal models to study access of CoQ10 across the BBB and subsequent neuronal metabolism has, therefore, been questioned, and there is an argument, perhaps surprisingly, that in vitro model systems (particularly 3D cellular systems) may be more appropriate. In this article, we have therefore reviewed the role of model systems to study access of CoQ10 across the BBB, as well as the role of such systems in studying the role of CoQ10 in aspects of neuronal metabolism, such as mitochondrial and lysosomal function. Relevant articles in the peer-reviewed literature were identified using the Medline database and the keywords listed in the abstract.

## 2. Blood–Brain Barrier Model Systems

BBB model systems comprise in vitro cell culture models and in vivo animal-based models. In vitro BBB models include 2D and 3D cellular systems. In the so-called Transwell 2D model, a monolayer of endothelial cells is cultured on a permeable membrane with a small semi-permeable insert, separating the system into upper (luminal or blood) compartment and lower (abluminal or parenchymal) compartments. While this model is useful for evaluating the permeability of medicinal drugs across the BBB, the monolayer of cells does not replicate the complex 3D structure of the BBB in vivo, and the static nature of the culture lacks the physiological shear stress from blood flow that is crucial for endothelial cell differentiation and maintenance of BBB properties. The physiological relevance of this model system can be improved by including other cell types (e.g., astrocytes, neurons, or pericytes), as well as incorporating a system to perfuse the endothelium, thereby providing shear stress [4].

In the brain, the BBB is a tubular structure, which is not accurately replicated using 2D models; 3D models include the different cell types (brain endothelial cells, pericytes, and astrocytes) that form the neurovascular unit (NVU). Hydrogel-based models use a supporting collagen matrix to create a 3D environment in which cells can grow and self-assemble into tubular structures, similar to the in vivo microvasculature. Microfluidic BBB-on-a-chip models are lab-on-a-chip platforms that integrate NVU cells within microchannels to create a perfusable network, allowing for dynamic studies of barrier function, fluid flow, and responses to external stimuli. These systems represent models of increasing complexity; 2D models are useful for initial screening and evaluating basic barrier integrity, whereas multicellular models provide a more comprehensive system to study BBB functions, including size-selective transport, the activity of P-glycoprotein (P-gp) efflux pumps, and the functionality of specific transport systems such as the transferrin receptor [5].

## 3. BBB Model Systems and CoQ10

**Figure 1:**
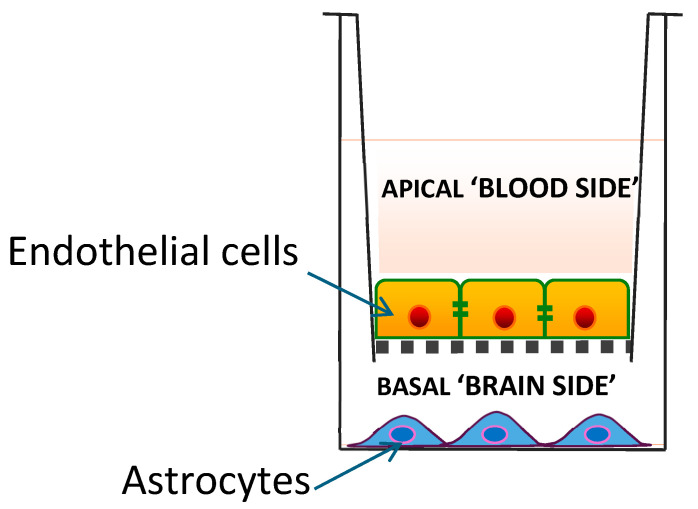
A 2D porcine endothelial monolayer blood–brain barrier model.

The only in vitro BBB model system used to date to investigate access by CoQ10 is the 2D porcine endothelial monolayer cell model described by Wainwright et al. [6] (Figure 1). In this model system, CoQ10 is transported in the apical-to-basal direction (i.e., blood-to-brain side) via lipoprotein-associated transcytosis, interacting with the SR-B1 (Scavenger Receptor) and RAGE (Receptor for Advanced Glycation End products) receptors. However, it is simultaneously effluxed back to the blood side via the LDLR (low-density lipoprotein receptor) transporter, leading to no net accumulation of CoQ10 in the brain. When a deficiency of CoQ10 in the model BBB is induced using para-aminobenzoic acid (PABA), the BBB becomes leakier, with disrupted tight junctions and poorer integrity; this, in turn, results in increased net CoQ10 transport from the blood side to the brain side.

Some studies in BBB model systems have been reported using the CoQ10 analogues idebenone and mitoquinone [7]. However, although these analogues have antioxidant activity in common with CoQ10, their other intracellular functions differ due to their varying chemical structures; for example, mitoquinone cannot transfer electrons from Complex I to Complex III in the mitochondrial electron transport chain (ETC) during oxidative phosphorylation.

The suitability of in vivo rodent-based BBB models to extrapolate data for BBB accessibility of CoQ10 in humans has been questioned because of the differences in the BBB structure and transporter expression, and there is some evidence that the BBB in rodents may be more permeable. For example, Uchida et al. [8] reported that protein expression levels of transporters and receptors in the BBB of humans were remarkably smaller than those in the BBB of rats. Similarly, supplementation with CoQ10 in mouse models of Alzheimer’s disease (AD) has been reported to exhibit beneficial effects in reduced oxidative stress and beta-amyloid plaque levels and improved cognitive function [9]. However, in a randomised clinical trial in which 70 patients with mild-to-moderate AD were treated with CoQ10 (400 mg; three times/day) for 16 weeks, no clinical benefit or significant effect on the CSF biomarkers for AD (amyloid-beta and tau protein levels) was reported [10]. These data suggest a disparity between BBB accessibility in rodents and in humans. In another study, Matthews et al. [11] reported a 30% increase in cerebral cortex CoQ10 and coenzyme Q9 (CoQ9, the predominant ubiquinone species in rats) following oral supplementation of 12-month-old Sprague-Dawley rats with CoQ10 (200 mg/kg) for 2 months. In addition, Smith et al. [12] reported significant (*p* < 0.01) increases in brain levels of CoQ10 and CoQ9 following supplementation with high-dose (1000–5000 mg/kg) CoQ10 in a mouse model of Huntington’s disease. However, it is uncertain from these studies whether the degree of cerebral uptake of CoQ10 would be sufficient to replenish cellular levels of this quinone in a CoQ10 deficiency state. Given the suggested limited uptake of CoQ10 across the BBB, therapeutic strategies that may enhance this transport, such as the use of LDLR inhibitors, or interventions to stimulate luminal activity of SR-B1 transporters may be appropriate to ensure sufficient exogenous CoQ10 is available in the cerebral interstitial fluid for metabolically compromised neurons [6].

## 4. Supplementation with Ubiquinone Versus Ubiquinol Forms of CoQ10: Comparative Bioavailability and BBB Access

CoQ10 is transported in the blood circulation and bound to low-density lipoprotein and very low-density lipoprotein–cholesterol, irrespective of the initial dietary form (ubiquinone or ubiquinol). Accordingly at some point, dietary CoQ10 in the oxidised ubiquinone form must, therefore, be reduced to the ubiquinol form, and this was thought to take place during the absorption process into enterocytes [2]. It was, therefore, postulated that supplemental CoQ10 already in ubiquinol form would facilitate the absorption process, resulting in improved bioavailability. However, research carried out by the late Dr William Judy demonstrated that under conditions simulating the environment of the stomach and small intestine in vitro, supplemental ubiquinol is largely oxidised to ubiquinone prior to entry into enterocytes [2]. A standard gelatine capsule dissolves in the stomach in approximately 10 min, releasing the ubiquinol into the stomach environment. The time for ingested material to remain in the stomach can be quite variable, but a reasonable estimate would be about 4 h [13]. If the capsule is taken with food, free ubiquinol will be distributed among other stomach contents by the churning action of the stomach. The stomach represents an oxidising environment [14], so ubiquinol would be oxidised to ubiquinone during this period, before ever reaching the small intestine and subsequent enterocyte absorption.

In addition, in vivo studies supplementing ubiquinol in dogs similarly showed oxidation of the latter to ubiquinone prior to enterocyte absorption, with the subsequent conversion of ubiquinone back to ubiquinol following the passage from enterocytes into the lymphatic system [15,16]. CoQ10 that has been subjected to a patented crystal modification process shows improved bioavailability [2]. In a clinical study, Lopez-Lluch and colleagues [17] reported that the bioavailability of a ubiquinol form of a particular CoQ10 supplement was approximately twice that of ubiquinone that had not been subjected to thermal crystal modification, but was only 52% of that of ubiquinone that had been subjected to thermal crystal modification. Based on the above, there is, therefore, no rationale why the bioavailability (i.e., access to the blood circulation) of the ubiquinol form of CoQ10 should be superior to that of the ubiquinone form, which is a common misconception.

It has also been suggested that in human subjects, the reduced form of CoQ10 (ubiquinol) may be able to access the BBB, while the oxidised form of CoQ10 (ubiquinone) is not able to cross the BBB. Thus, following oral supplementation with ubiquinol, Mitsui et al. [18] reported increased levels of ubiquinol in the CSF of patients with multiple system atrophy (MSA). There are essentially two barriers preventing access of CoQ10 into the human brain: the BBB and the brain CSF barrier (BCSFB). The BCSFB is relatively leaky compared to the BBB, and access of CoQ10 (in either ubiquinone or ubiquinol form) into the CSF via the BCSFB does not equate to access of CoQ10 into the brain parenchyma—another common misconception [19]. To date there have been no clinical studies in which orally administered CoQ10 has been unambiguously shown to directly cross the BBB and access the human brain. Whether CoQ10, in either ubiquinone or ubiquinol forms, can access the BBB in humans, and the mechanism by which CoQ10 is then distributed within brain cells, has yet to be elucidated.

In view of the potential limited transport of CoQ10 across the BBB, other strategies may be appropriate to restore cerebral CoQ10 status. It has recently been reported that the intermediates 4-hydroxymandelate and 4-hydroxybenzoate may have the potential to cross the BBB and restore cerebral CoQ10 status, serving as a potential treatment for primary CoQ10 deficiencies [20]. These intermediates have been investigated in mouse models of mitochondrial disease and were reported to increase cerebral ubiquinone status (CoQ10 plus CoQ9), although no studies have yet investigated the effect of these precursor molecules on CoQ10-deficient human neuronal cells.

Currently, the level of plasma CoQ10 that may have therapeutic potential in the treatment of disease is uncertain. In a study by Langsjoen and Langsjoen [21], a blood concentration of approximately 4.1 mM was required before any therapeutic benefit was identified in patients with congestive heart failure. No studies to date have assessed this parameter in patients with CoQ10 deficiency, although a study by Lopez and colleagues [22] reported an improvement in bioenergetic status, as indicated by an increased ATP/ADP ratio and normalisation of cellular oxidative stress in CoQ10-deficient fibroblasts following 7 days of supplementation with 5 mM CoQ10. Interestingly, a comparative circulatory level of CoQ10 was associated with a slowing in the progressive deterioration of function in Parkinson’s disease patients in a randomised, double-blind, placebo-controlled study by Shults and colleagues [23], which assessed the effect of CoQ10 supplementation in early-stage Parkinson’s disease patients.

## 5. BBB, CoQ10, and Vitamin E

Co-administration of CoQ10 and vitamin E has been reported to be of synergistic benefit in both preclinical and clinical studies of neurological disorders [24,25]. Such studies raise the question of how vitamin E accesses the brain in comparison to CoQ10.

Access of vitamin E across the BBB involves carrier-mediated transport, which is mediated via the SR-B1 receptor uptake of HDL complexed alpha tocopherol, together with the protein afamin; the inter- and intra-cellular distribution of vitamin E is then controlled by the tocopherol transfer protein. As the uptake of vitamin E across the BBB involves the same SR-B1 receptor as that involved in the uptake of CoQ10, one might question whether restricted brain access for either substance might occur as a result of competition for the same transporter. Indeed, in the porcine in vitro BBB model, alpha tocopherol was found to increase basal-to-apical transport of CoQ10 in control conditions, i.e., towards the blood side. In the presence of alpha-tocopherol, CoQ10 transport towards the blood side dominated in the PABA-induced CoQ10-deficient BBB model [6]. If this translates to clinical CoQ10 deficiency, then alpha-tocopherol co-administration with CoQ10 supplements would tend to reduce CoQ10 delivery towards the brain, the opposite of the desired effect.

In this regard, it is of note that in a Phase II clinical study, CoQ10 in daily doses of 300 mg, 600 mg, or 1200 mg administered to Parkinson’s disease patients resulted in a significant slowing of functional decline [23]. However, in a Phase III study, daily doses of CoQ10 (1200 mg or 2400 mg) administered to Parkinson’s disease patients, together with a daily dose of 1200 IU of vitamin E, had no significant symptomatic benefit, in contrast to the outcome of the Phase II study [26]. These data therefore suggest the possibility that co-administration of a high dose of vitamin E could have inhibited access to the brain for CoQ10 via competition for a shared carrier. It is also possible that vitamin E may interfere with the absorption of co-administered CoQ10 from the digestive tract via competition for the same transporter (putatively the cholesterol transporter NPC1L1 (Niemann-Pick C1 Like 1) [13,27].

## 6. Neuronal Cell Model Systems and CoQ10

Model systems for studying the metabolism of CoQ10 in neurons typically use human SH-SY5Y neuronal cell lines in 2D or 3D culture systems. Para-aminobenzoic acid (PABA), a competitive inhibitor of the COQ2 enzyme in the CoQ10 biosynthetic pathway, can be used to induce CoQ10 deficiency to study the effect of CoQ10 deficiency on neuronal metabolism. This type of model system has been used to elucidate the action of supplementary CoQ10 in neurological disorders of CoQ10 deficiency and phenylketonuria, as described below. At present, the concentration of plasma or cerebral spinal fluid CoQ10 that may have therapeutic potential in the treatment of neurological disorders associated with mitochondrial dysfunction and oxidative stress has yet to be determined. In vitro studies utilising CoQ10-deficient human fibroblasts have reported an improvement in bioenergetic status and normalisation of cellular oxidative stress following treatment with CoQ10 at a concentration of 5 μM. However, it is uncertain whether this concentration of CoQ10 would be physiologically achievable in human neurons in vivo, i.e., whether CoQ10 is able to cross the human BBB, and have therapeutic potential in the treatment of the neurological symptoms associated with metabolic disease.

To investigate this question at the biochemical level, we evaluated the effect of treatment with 5 μM CoQ10 on a human neuronal cell model of CoQ10 deficiency [28]. The human neuronal cell model of CoQ10 deficiency was established using human SH-SY5Y neuronal cells. In the CoQ10-deficient neuronal cells, supplementing with 5 uM CoQ10 was effective at significantly reducing mitochondrial oxidative stress (*p* < 0.0005) to below control levels. In contrast, CoQ10 supplementation at concentrations of either 5 or 10 uM was only partially effective at restoring MRC enzyme activities to control levels, with complex II/III activity being significantly (*p* < 0.05) increased to 82.5%, complex I to 71.1%, and IV to 77.7% of control levels following treatment with 10 uM CoQ10. Treatment with 5 uM CoQ10 was also found to decrease the lysosomal pH of the CoQ10-deficient neurons from 6.2 to 4.4, which is within the pH range of control cells [29]. The results of this study have indicated that although cellular and mitochondrial oxidative stress in a neuronal cell model of CoQ10 deficiency appear to be attenuated following CoQ10 supplementation with 5 uM CoQ10, MRC enzyme activities were partially refractory to treatment, and supplementation with doses of CoQ10 > 10 uM may be required to restore MRC enzyme activities to control levels. However, given the suggested limited uptake of CoQ10 across the human blood–brain barrier, therapeutic strategies that may enhance this transport such as the use of LDLR (low-density lipoprotein related protein −1 receptor) inhibitors, or interventions to stimulate luminal activity of SR-B1 (Scavenger Receptor B1) transporters may be appropriate to ensure sufficient exogenous CoQ10 is available within the interstitial fluid for metabolically compromised neurons [6]. This study indicates the potential requirement for high-dose CoQ10 supplementation to treat the neurological presentations of CoQ10 deficiency, although whether this will be able to reach the CoQ10-deficient neurons is still uncertain.

Studies using this model system have demonstrated the key role of CoQ10 for maintaining the acidic pH (quantified via the fluorescent probes Lysotracker or LysoSensor) within neuronal lysosomes, which is essential for their role in the degradation of cellular waste products. CoQ10 acts as a proton carrier at the lysosomal membrane, and its deficiency disrupts this process, leading to an increase in lysosomal pH and potentially impairing lysosomal and autophagy functions. Administration of CoQ10 results in restoration of normal lysosomal pH and neuronal lysosomal function [29]. Neurons are particularly susceptible to a build-up of cellular waste products resulting from lysosomal dysfunction, in turn resulting in neurological lysosomal storage disorders including Niemann–Pick disease, mucopolysaccharidosis (MPS), Batten’s disease, Gaucher’s disease, and Krabbe’s disease. The potential role of supplementary CoQ10 in the treatment of patients with lysosomal storage disorders is an area in the early stages of clinical research.

Model systems based on the human SH-SY5Y neuroblastoma cell line have also been used to elucidate the mechanism(s) of intracellular CoQ10 transport, as described in the following section of this article.

## 7. Model Systems to Study Intracellular Transport of CoQ10—Role of STARD7

CoQ10 is synthesised within mitochondria and must then be relocated to other subcellular and cell membranes. The mechanism(s) by which CoQ10 is transported within the cell is not completely understood but is thought to involve the lipid transfer protein STARD7 (steroidogenic acute regulatory protein related lipid transfer domain 7). The interaction between CoQ10 and STARD7 has been investigated in model systems using several types of human cell lines, including SH-SY5Y neuroblastoma and HEK293 cells. Using the latter model system, two key roles for STARD7 were identified: firstly, in the transport of phosphatidylcholine to mitochondria to support the biosynthesis of CoQ10, and secondly, to transport newly synthesised CoQ10 from mitochondria to subcellular and cellular membranes. The function of STARD7 was, in turn, shown to be regulated by the mitochondrial protease PARL, which releases the STARD7-CoQ10 complex from the mitochondria for transport within the cell. CRISPR/Cas9 technology to create STARD7 or PARL knockout HEK 293 cell lines was used to establish how the absence of these proteins affects CoQ10 production, transport, and mitochondrial health [30,31,32].

A model system based on the human liver cancer cell line HepG2 has been used to investigate the intracellular transport of CoQ10 by another type of lipid carrier, Saposin B, and its precursor prosaposin [33]. Saposin B transports newly synthesised CoQ10 from the mitochondria to other sub-cellular organelles for subsequent membrane incorporation, including lysosomes. The binding of CoQ10 to Saposin B is pH-dependent, such that the acidic internal pH of the lysosomes causes Saposin B to release the transported CoQ10 [34]. It is of note that Saposin B is also an intracellular carrier of vitamin E, suggesting the possibility of competition in the transport of CoQ10, as noted previously in this article [35].

Human neuronal model systems based on the neuroblastoma cell line SH-SY5Y have similarly been used to investigate the mechanism of intracellular CoQ10 transport by STARD7 and Saposin B, as well as by the Cqd1 protein, which promotes the export of CoQ10 from mitochondria [31]. It is also of note that human-cell-based model systems have been used to investigate vesicle-mediated intracellular transport of CoQ10, which operates in tandem with the lipid transporters above, particularly regarding transport to the endoplasmic reticulum and Golgi apparatus [32].

## 8. Model Systems for Studying Intranasal Administration of CoQ10

Intranasal drug delivery provides a mechanism by which drugs can be delivered directly into the brain via transport along the olfactory and trigeminal nerves, bypassing the BBB. While this route has been used for the administration of several prescription-type medicinal products relevant to the treatment of CNS disorders, to date, there have been no clinical studies relating to intranasal administration of CoQ10 [2]. Although animal (primarily rodent)-based models are available for studying intranasal drug administration, the applicability of such systems for studying the delivery of CoQ10 in humans has been questioned because of differences in nasal anatomy between rodents and humans [19]; there is, therefore, a rationale for the use of human-cell-based model systems for studying the intranasal delivery of CoQ10.

Cell-based model systems for studying intranasal drug delivery include cell lines or primary cell cultures derived from human nasal epithelium. Cell lines represent a simpler system in use, particularly for preliminary screening, but may be lacking in some functional characteristics. Primary cultures represent a more complex system to use but contain all the cell types found in the human nasal mucosa, including ciliated cells and mucus-producing goblet cells, and are used to study drug transport mechanisms [36]. No studies were identified to date in which this type of model system had been used to study the intranasal administration of CoQ10.

## 9. Model Systems for Studying Selenium Metabolism

One area of importance to CoQ10 metabolism, although sometimes overlooked, is the interaction between CoQ10 and selenium. As noted earlier in this article, CoQ10 exists in both oxidised (ubiquinone) and reduced (ubiquinol) forms. The normal functioning of CoQ10 requires the continual interconversion of ubiquinone and ubiquinol CoQ10 forms (approximately 1.4 cycles per second [37]). Contrary to the marketing literature provided by some supplement companies supplying CoQ10, the ubiquinol form of CoQ10 is, therefore, not more important than the ubiquinone form in cellular metabolism. The importance of selenium in the CoQ10 recycling process is in its role as an essential cofactor of the flavoenzyme thioredoxin reductase, which converts ubiquinone to ubiquinol to help facilitate the continual recycling of CoQ10. It is of note that thioredoxin reductase is not the only enzyme converting ubiquinone to ubiquinol, but is one of the most important [37]. Thioredoxin reductase uses NADPH as an electron donor, transferring electrons to CoQ10; the amino acid derivative, selenocysteine, forms part of the active site and is vital for the activity of this enzyme. This interaction at the cellular level explains the synergistic action of CoQ10 and selenium reported in clinical studies [38].

In general terms, both 2D and 3D cell cultures have been used as model systems to study selenium metabolism in human cells, including embryonic kidney cells (HEK 293), hippocampal progenitor cells (HPCs), and colon cancer cells (Caco 2, HT 29). Specifically, regarding the brain, model systems using human cells to study how selenium crosses the human BBB have used brain microvascular endothelial cells in static transwell systems, as well as BBB-on-a-chip models. Using such model systems, it has been shown that selenium (transported in the form selenoprotein P) enters cells via a receptor-mediated endocytosis pathway involving the low-density lipoprotein receptor ApoER2; this process also allows selenium (in selenoprotein P form) to access the BBB [39].

Several types of model systems have been used to study the interaction between CoQ10 and selenium, including selenium-deficient cell cultures. Cell lines grown in selenium-deficient media mimic the effect of dietary selenium deficiency on mitochondrial function and oxidative stress. Cell types with high energy or antioxidant demands, such as liver cells, are particularly sensitive to selenium deficiency; human hepatocytes cultured in selenium-deficient media have reduced levels of thioredoxin reductase, as well as the antioxidant selenoenzyme glutathione peroxidase [40].

## 10. Discussion

For obvious reasons, the ability to study CoQ10 metabolism in the human brain in vivo is extremely limited. Non-invasive imaging techniques such as phosphorus-31 magnetic resonance spectroscopy can be used to assess brain energy metabolism in vivo. However this technique evaluates the effect of CoQ10, for example, on mitochondrial function and ATP generation (by measuring phosphorylated compounds such as phosphocreatine), rather than measuring CoQ10 levels directly. At present there are no suitable techniques available for monitoring CoQ10 directly in the human brain in vivo, particularly whether it can access the human BBB. This, in turn, raises the question of the most suitable model systems relevant to CoQ10 metabolism in the human brain; as noted earlier in this article, perhaps surprisingly, there is an argument that in vitro model systems based on human cell lines are more appropriate than studies in animal models, particularly regarding BBB accessibility, although data obtained from such studies needs to be interpreted in context. The advantages and disadvantages of the various cell-line-based BBB model systems described earlier in this article are summarised in Table 1.

The model systems described in this review have been used to further investigate the access of CoQ10 across the BBB and its metabolism within neurons, including mechanisms of intracellular transport. A 2D BBB model system based on endothelial cells has demonstrated the transport of CoQ10 in the apical-to-basal direction (i.e., blood-to-brain side) via lipoprotein-associated transcytosis, interacting with the SR-B1 and RAGE receptors. However, CoQ10 is simultaneously effluxed back to the blood side via the LDLR transporter, leading to no net accumulation of CoQ10 in the brain. Similarly a 2D neuronal model system based on CoQ10-deficient human SH-SY5Y cells has demonstrated the beneficial effects (in terms of improved mitochondrial function, reduced oxidative stress, and improved lysosomal function) of CoQ10 supplementation. Model systems based on the human SH-SY5Y neuroblastoma cell line have also been used to elucidate the mechanism(s) of intracellular CoQ10 transport; however, there are still many aspects of CoQ10 intracellular transport (and other transport situations) that require further elucidation, but the known CoQ10 transporters identified to date are summarised in Table 2.

**Figure 2:**
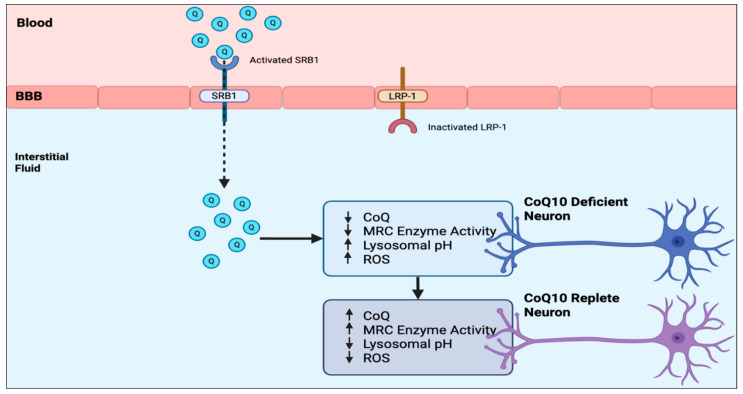
Strategies to enhance CoQ_10_ transport across the human BBB and its effects on CoQ_10_-deficient neurons. BBB—blood–brain barrier; Q—CoQ10; CoQ—CoQ10; MRC—mitochondrial respiratory chain; SRB1—Scavenger Receptor class B type 1; LRP-1—low-density lipoprotein receptor-related protein 1; ROS—Reactive Oxygen Species.

Finally, based on the model systems described in this article, a potential strategy to enhance CoQ10 transport across the human BBB and subsequently improve neuronal metabolism, based on activation of SRB1 and inhibition of LRP-1, respectively, is shown in Figure 2. With regard to SRB1 and LRP-1, broad-spectrum activation or inhibition, respectively, is not considered advisable because of their multiple functions. However, SRB1 and LRP-1 still constitute potential targets for improving CoQ10 BBB access using more specific activators or inhibitors of their interaction with CoQ10, and this remains an area for future research.

## Figures and Tables

**Table 1:** Comparative summary of cell-line-based BBB model systems for the study of CoQ10 metabolism.

Parameter	2D Systems	3D Systems
Structure	Flat monolayer of endothelial cells	Tubular structure comprising several cell types (endothelial cells, pericytes, astrocytes)
Setup	Easy to establish, cost-effective, suitable for high-throughput studies	More complex and expensive to establish, lower throughput
Physiological relevance	More limited physiological relevance, suitable for initial screening studies	More physiologically relevant, more realistic model of BBB function

**Table 2:** Summary of characteristics of CoQ10 transporters.

Transporter	Function
STARD7	Intracellular transport of CoQ10 from mitochondria to other subcellular organelles
Saposin-B	Intracellular transport of CoQ10 from mitochondria to other subcellular organelles
Cqd1	Export of CoQ10 from mitochondria
Cqd2	Import of CoQ10 into mitochondria
P-glycoprotein	Export of CoQ10 out of cells
NPC1L1 protein	Transport of CoQ10 from intestine into enterocytes
LDL cholesterol, VLDL cholesterol	Transport of CoQ10 in blood circulation

LDL—low-density lipoprotein; VLDL—very low-density lipoprotein; NPC1L1—Niemann–Pick C1Like1 protein; Cqd1—coenzyme Q distribution protein 1; Cqd2—coenzyme Q distribution protein 2.

## Data Availability

No new data were created or analyzed in this study. Data sharing is not applicable to this article.

## Abbreviations

The following abbreviations are used in this manuscript:

CoQ10	Coenzyme Q10
BBB	Blood–brain barrier
MRC	Mitochondrial respiratory chain
SRB1	Scavenger receptor class B type 1
LRP-1	Low-density lipoprotein receptor-related protein 1
ROS	Reactive oxygen species
BCSFB	Brain CSF barrier

## References

[1] Mortensen S.A., Rosenfeldt F., Kumar A., Dolliner P., Filipiak K.J., Pella D., Alehagen U., Steurer G., Littarru G.P. (2014). The effect of coenzyme Q10 on morbidity and mortality in chronic heart failure: Results from Q-SYMBIO: A randomized double-blind trial. JACC Heart Fail..

[2] Mantle D., Lopez-Lluch G., Hargreaves I.P. (2023). Coenzyme Q10 Metabolism: A Review of Unresolved Issues. Int. J. Mol. Sci..

[3] Mantle D., Millichap L., Castro-Marrero J., Hargreaves I.P. (2023). Primary Coenzyme Q10 deficiency: An update. Antioxidants.

[4] Stone N.L., England T.J., O’Sullivan S.E. (2019). A Novel Transwell Blood Brain Barrier Model Using Primary Human Cells. Front. Cell Neurosci..

[5] Pérez-López A., Torres-Suárez A.I., Martín-Sabroso C., Aparicio-Blanco J. (2023). An overview of in vitro 3D models of the blood-brain barrier as a tool to predict the in vivo permeability of nanomedicines. Adv. Drug Deliv. Rev..

[6] Wainwright L., Hargreaves I.P., Georgian A.R., Turner C., Dalton R.N., Abbott N.J., Heales S.J.R., Preston J.E. (2020). CoQ10 Deficient Endothelial Cell Culture Model for the Investigation of CoQ10 Blood-Brain Barrier Transport. J. Clin. Med..

[7] Montenegro L., Trapani A., Latrofa A., Puglisi G. (2012). In vitro evaluation on a model of blood brain barrier of idebenone-loaded solid lipid nanoparticles. J. Nanosci. Nanotechnol..

[8] Uchida Y., Yagi Y., Takao M., Tano M., Umetsu M., Hirano S., Usui T., Tachikawa M., Terasaki T. (2020). Comparison of absolute protein abundances of Transporters and receptors among blood-brain barriers at different cerebral regions and the blood-spinal cord barrier in humans and rats. Mol. Pharm..

[9] Dumont M., Kipiani K., Yu F., Wille E., Katz M., Calingasan N.Y., Gouras G.K., Lin M.T., Beal M.F. (2011). Coenzyme Q10 decreases amyloid pathology and improves behaviour in a transgenic mouse model of Alzheimer’s disease. J. Alzheimer’s Dis..

[10] Galasko D.R., Peskind E., Clark C.M., Quinn J., Ringman J.M., Jicha G.A., Cotman C., Cottrell B., Montine T.J., Thomas R.G. (2012). Antioxidants for Alzheimer disease: A randomized clinical trial with cerebrospinal fluid biomarker measures. Arch. Neurol..

[11] Matthews R.T., Yang L., Browne S., Baik M., Beal M.F. (1998). Coenzyme Q10 administration increases brain mitochondrial concentrations and exerts neuroprotective effects. Proc. Natl. Acad. Sci. USA.

[12] Smith K.M., Matson S., Matson W.R., Cormier K., Del Signore S.J., Hagerty S.W., Stack E.C., Ryu H., Ferrante R.J. (2006). Dose ranging and efficacy study of high-dose coenzyme Q10 formulations in Huntington’s disease mice. Biochem. Biophys. Acta.

[13] Mantle D., Dybring A. (2020). Bioavailability of Coenzyme Q10: An overview of the absorption process and subsequent metabolism. Antioxidants.

[14] Even A., Minderhoud R., Torfs T., Leonardi F., van Heusden A., Sijabat R., Firfilionis D., Castro Miller I.D., Rammouz R., Teichmann T. (2025). Measurements of Redox Balance along the Gut Using a Miniaturised Ingestible Sensor. Nat. Electron..

[15] Judy W.V. (2021). The instability of the lipid-soluble antioxidant ubiquinol: Part 1-lab studies. Integr. Med..

[16] Judy W.V. (2021). The instability of the lipid-soluble antioxidant ubiquinol: Part 2-dog studies. Integr. Med..

[17] Lopez-Lluch G., Del Pozo-Cruz J., Sanchez-Cuesta A., Cortes-Rodriguez A.B., Navas P. (2019). Bioavailability of coenzyme Q10 supplements depends on carrier lipids and solubilization. Nutrition.

[18] Mitsui J., Koguchi K., Momose T., Takahashi M., Matsukawa T., Yasuda T., Tokushige S.I., Ishiura H., Goto J., Nakazaki S. (2017). Three-Year follow-up of high dose ubiquinol supplementation in a case of familial multiple system atrophy with compound heterozygous COQ2 mutations. Cerebellum.

[19] Mantle D., Hargreaves I. (2025). Coenzyme Q10 and the Blood-Brain Barrier: An Overview. J. Clin. Med..

[20] Shi G., Miller C., Kuno S., Rey Hipolito A.G., El Nagar S., Riboldi G.M., Korn M., Tran W.C., Wang Z., Ficaro L. (2025). Coenzyme Q headgroup intermediates can ameliorate a mitochondrial encephalopathy. Nature.

[21] Langsjoen P.H., Langsjoen A.M. (1999). Overview of the use of CoQ10 in cardiovascular disease. Biofactors.

[22] López L.C., Quinzii C.M., Area E., Naini A., Rahman S., Schuelke M., Salviati L., Dimauro S., Hirano M. (2010). Treatment of CoQ(10) deficient fibroblasts with ubiquinone, CoQ analogs, and vitamin C: Time- and compound-dependent effects. PLoS ONE.

[23] Shults C.W., Oakes D., Kieburtz K., Beal M.F., Haas R., Plumb S., Juncos J.L., Nutt J., Shoulson I., Carter J. (2002). Effects of coenzyme Q10 in early Parkinson disease: Evidence of slowing of the functional decline. Arch. Neurol..

[24] Kasparová S., Sumbalová Z., Bystrický P., Kucharská J., Liptaj T., Mlynárik V., Gvozdjáková A. (2006). Effect of coenzyme Q10 and vitamin E on brain energy metabolism in the animal model of Huntington’s disease. Neurochem. Int..

[25] Cooper J.M., Korlipara L.V., Hart P.E., Bradley J.L., Schapira A.H. (2008). Coenzyme Q10 and vitamin E deficiency in Friedreich’s ataxia: Predictor of efficacy of vitamin E and coenzyme Q10 therapy. Eur. J. Neurol..

[26] Beal M.F., Parkinson Study Group (2014). A randomized clinical trial of high-dosage coenzyme Q10 in early Parkinson disease: No evidence of benefit. JAMA Neurol..

[27] Narushima K., Takada T., Yamanashi Y., Suzuki H. (2008). Niemann-pick C1-like 1 mediates alpha-tocopherol transport. Mol. Pharmacol..

[28] Duberley K.E., Heales S.J., Abramov A.Y., Chalasani A., Land J.M., Rahman S., Hargreaves I.P. (2014). Effect of Coenzyme Q(10) supplementation on mitochondrial electron transport chain activity and mitochondrial oxidative stress in Coenzyme Q(10) deficient human neuronal cells. Int. J. Biochem. Cell Biol..

[29] Heaton R.A., Heales S., Rahman K., Sexton D.W., Hargreaves I. (2020). The Effect of Cellular Coenzyme Q10 Deficiency on Lysosomal Acidification. J. Clin. Med..

[30] Saita S., Tatsuta T., Lampe P.A., König T., Ohba Y., Langer T. (2018). PARL partitions the lipid transfer protein STARD7 between the cytosol and mitochondria. EMBO J..

[31] Deshwal S., Onishi M., Tatsuta T., Bartsch T., Cors E., Ried K., Lemke K., Nolte H., Giavalisco P., Langer T. (2023). Mitochondria regulate intracellular coenzyme Q transport and ferroptotic resistance via STARD7. Nat. Cell Biol..

[32] Guile M.D., Jain A., Anderson K.A., Clarke C.F. (2023). New Insights on the Uptake and Trafficking of Coenzyme Q. Antioxidants.

[33] Kashiba M., Oizumi M., Suzuki M., Sawamura Y., Nagashima K., Yoshimura S., Yamamoto Y. (2014). Prosaposin regulates coenzyme Q10 levels in HepG2 cells, especially those in mitochondria. J. Clin. Biochem. Nutr..

[34] Jin G., Kubo H., Kashiba M., Horinouchi R., Hasegawa M., Suzuki M., Jin G., Kubo H., Kashiba M., Horinouchi R. (2008). Saposin B is a human coenzyme q10-binding/transfer protein. J. Clin. Biochem. Nutr..

[35] Jin G., Horinouchi R., Sagawa T., Orimo N., Kubo H., Yoshimura S., Fujisawa A., Kashiba M., Yamamoto Y. (2008). Coenzyme Q10-Binding/Transfer Protein Saposin B Also Binds Gamma-Tocopherol. J. Clin. Biochem. Nutr..

[36] Boyuklieva R., Zagorchev P., Pilicheva B. (2023). Computational, In Vitro, and In Vivo models for nose-to-brain drug delivery studies. Biomedicines.

[37] Mantle D., Dewsbury M., Hargreaves I.P. (2024). The Ubiquinone-Ubiquinol Redox Cycle and Its Clinical Consequences: An Overview. Int. J. Mol. Sci..

[38] Alehagen U., Johansson P., Björnstedt M., Rosén A., Dahlström U. (2013). Cardiovascular mortality and N-terminal-proBNP reduced after combined selenium and coenzyme Q10 supplementation: A 5-year prospective randomized double-blind placebo-controlled trial among elderly Swedish citizens. Int. J. Cardiol..

[39] Solovyev N., Drobyshev E., Blume B., Michalke B. (2021). Selenium at the neural barriers: A review. Front. Neurosci..

[40] Saito Y., Yoshida Y., Akazawa T., Takahashi K., Niki E. (2003). Cell death caused by selenium deficiency and protective effect of antioxidants. J. Biol. Chem..

